# Wollamide Cyclic Hexapeptides Synergize with Established and New Tuberculosis Antibiotics in Targeting Mycobacterium tuberculosis

**DOI:** 10.1128/spectrum.00465-23

**Published:** 2023-06-08

**Authors:** Rachel F. Rollo, Giorgia Mori, Timothy A. Hill, Doris Hillemann, Stefan Niemann, Susanne Homolka, David P. Fairlie, Antje Blumenthal

**Affiliations:** a Frazer Institute, The University of Queensland, Brisbane, Queensland, Australia; b Institute for Molecular Bioscience, The University of Queensland, Brisbane, Queensland, Australia; c Australian Research Council Centre of Excellence for Innovations in Peptide and Protein Science, Institute for Molecular Bioscience, The University of Queensland, Brisbane, Queensland, Australia; d National and WHO Supranational Reference Center for Mycobacteria, Research Center Borstel, Leibniz Lung Center, Borstel, Germany; e Molecular and Experimental Mycobacteriology, Priority Area Infections, Research Center Borstel, Leibniz Lung Center, Borstel, Germany; f German Center for Infection Research (DZIF), Partner Site Hamburg-Lübeck-Borstel-Riems, Borstel, Germany; Johns Hopkins University School of Medicine

**Keywords:** *Mycobacterium tuberculosis*, antimicrobial combinations, multidrug resistance

## Abstract

Shorter and more effective treatment regimens as well as new drugs are urgent priorities for reducing the immense global burden of tuberculosis (TB). As treatment of TB currently requires multiple antibiotics with diverse mechanisms of action, any new drug lead requires assessment of potential interactions with existing TB antibiotics. We previously described the discovery of wollamides, a new class of *Streptomyces*-derived cyclic hexapeptides with antimycobacterial activity. To further assess the value of the wollamide pharmacophore as an antimycobacterial lead, we determined wollamide interactions with first- and second-line TB antibiotics by determining fractional inhibitory combination index and zero interaction potency scores. *In vitro* two-way and multiway interaction analyses revealed that wollamide B1 synergizes with ethambutol, pretomanid, delamanid, and para-aminosalicylic acid in inhibiting the replication and promoting the killing of phylogenetically diverse clinical and reference strains of the Mycobacterium tuberculosis complex (MTBC). Wollamide B1 antimycobacterial activity was not compromised in multi- and extensively drug-resistant MTBC strains. Moreover, growth-inhibitory antimycobacterial activity of the combination of bedaquiline/pretomanid/linezolid was further enhanced by wollamide B1, and wollamide B1 did not compromise the antimycobacterial activity of the isoniazid/rifampicin/ethambutol combination. Collectively, these findings add new dimensions to the desirable characteristics of the wollamide pharmacophore as an antimycobacterial lead compound.

**IMPORTANCE** Tuberculosis (TB) is an infectious disease that affects millions of people globally, with 1.6 million deaths annually. TB treatment requires combinations of multiple different antibiotics for many months, and toxic side effects can occur. Therefore, shorter, safer, more effective TB therapies are required, and these should ideally also be effective against drug-resistant strains of the bacteria that cause TB. This study shows that wollamide B1, a chemically optimized member of a new class of antibacterial compounds, inhibits the growth of drug-sensitive as well as multidrug-resistant Mycobacterium tuberculosis isolated from TB patients. In combination with TB antibiotics, wollamide B1 synergistically enhances the activity of several antibiotics, including complex drug combinations that are currently used for TB treatment. These new insights expand the catalogue of the desirable characteristics of wollamide B1 as an antimycobacterial lead compound that might inspire the development of improved TB treatments.

## INTRODUCTION

Tuberculosis (TB) is a leading cause of mortality due to a single infectious agent. In 2021 alone, it caused 1.6 million deaths and 10.6 million new cases of active TB globally, with one-quarter of the world’s population estimated to be latently infected (LTBI) with Mycobacterium tuberculosis complex (MTBC) strains (1–4). The significant adverse impact of the COVID-19 pandemic on recent progress in reducing the global TB burden has accentuated the urgent need for improved TB prevention and treatment (1, 5).

Treatment of active TB disease requires an extensive course of multiple antibiotics. Standard treatment for drug-sensitive (DS)-TB involves 2 months of isoniazid, rifampicin, pyrazinamide, and ethambutol, followed by 4 months of isoniazid and rifampicin (6). However, drug-resistant (DR)-TB poses significant challenges to treatment and is viewed not only as a public health concern but also as a threat to global security (1). Multidrug-resistant (MDR)-TB strains are MTBC strains with resistance to at least isoniazid and rifampicin, while extensively drug-resistant (XDR)-TB is defined as rifampicin-resistant or MDR-TB with additional resistance to any fluoroquinolone and at least one additional group A drug, i.e., bedaquiline or linezolid (7). Treatment of DR-TB requires significantly longer therapy (up to 20 months) with more complex drug regimens that are commonly associated with significant side effects (6), though recently, an all-oral regimen of bedaquiline, pretomanid, and linezolid (BPaL) was approved for treatment of MDR- and XDR-TB, with a treatment duration of 6 to 9 months (8, 9). The threat posed by DR-TB is exacerbated by treatment noncompliance due to toxic side effects of the available agents, limited accessibility to adequate treatment and surveillance in some regions, and evolution toward increasingly DR-TB strains (1, 10, 11). Therefore, new antibiotics are urgently needed for shorter and safer treatment of drug-sensitive and drug-resistant TB.

Progression of newly discovered antimicrobials toward development of new antibiotics is challenging, and in the case of TB, the complexity of this process is amplified by the requirement for new antibiotics to be integrated into existing or new drug combination regimens (11–13). A detailed understanding of the activity of new lead compounds in combination with existing TB antibiotics is a critical early step during the preclinical evaluation of candidate antitubercular agents. We previously reported the discovery of wollamides, cyclic hexapeptides originally isolated from a soil *Streptomyces* sp., MST-11508, which exhibits anti-mycobacterial activity (14). Wollamides inhibit growth of M. tuberculosis strains and Mycobacterium bovis BCG with low-μM MICs (MIC, 0.6 to 3.3 μM and 1.7 μM, respectively) (14–19). Our structure activity relationship-optimized synthetic wollamide, wollamide B1, showed desirable features of new antimycobacterial leads, including low cytotoxicity against mammalian cells (15–19), restriction of M. bovis BCG and M. tuberculosis H37Rv within infected macrophages (14, 15), and activity against clinical M. tuberculosis isolates that carry genetic mutations commonly associated with drug resistance (15). An important next step in assessing the value of wollamides as an antimycobacterial pharmacophore is the evaluation of wollamide interactions with first- and second-line TB antibiotics.

Here, we show that wollamide B1 exerts growth-inhibitory activity against pan-susceptible as well as DR, MDR, and XDR clinical MTBC strains. Using independent drug interaction models (20, 21), we observed that wollamide B1 synergizes with the newly approved nitroimidazoles pretomanid and delamanid, the first-line agent ethambutol, and the second-line agent para-aminosalicylic acid in inhibiting the growth of phylogenetically diverse clinical and reference MTBC strains. Moreover, wollamide B1 enhances the antimycobacterial activity of the recently introduced BPaL combination and does not antagonize the first-line regimen of isoniazid and rifampicin, with or without ethambutol. These data highlight that detailed exploration of the wollamide pharmacophore holds promise of identifying new leads effective against drug-sensitive and drug-resistant M. tuberculosis and for increasing the efficacy of TB antibiotic regimens.

## RESULTS

### Wollamide B1 inhibits the growth of phylogenetically diverse drug-sensitive and drug-resistant clinical M. tuberculosis isolates *in vitro*.

To expand our understanding of the wollamide antimycobacterial activity against MTBC strains, the growth inhibitory activity of wollamide B1 was assessed against MTBC clinical isolates representing different phylogenetic lineages (Table 1). Wollamide B1 inhibited the growth of pan-susceptible clinical MTBC strains of lineages 1, 2, and 4 in liquid cultures (MIC, 3.8 to 7.5 μM), comparable to the reference strain H37Rv (MIC, 3.8 μM) (Table 1).

**TABLE 1:** Wollamide B1 inhibits growth of phylogenetically diverse clinical M. tuberculosis isolates across lineages 1, 2, and 4 of the M. tuberculosis complex^*a*^

Isolate	Lineage	Genotype^*b*^	MIC (μM)	IC_50_ (μM)	Reference or source
H37Rv	4	Euro-American	3.8	0.6	NR-13648
4850/03	1.1.2	EAI	3.8	0.4	42
947/01	1.1.3	EAI	3.8	0.8	42
11406/08	1.2.1	EAI	7.5	0.8	42
4858/08	1.2.1	EAI	7.5	0.8	42
12594/02	2.2.1	Beijing	7.5	1.2	43
1500/03	2.2.1	Beijing	3.8	0.7	43
7253/02	2.2.2	Beijing	7.5	0.5	44
57/02	2.2.2	Beijing	7.5	0.4	44
4130/02	4.1.2.1	Haarlem	3.8	0.9	45
2336/02	4.1.2.1	Haarlem	7.5	1.4	45
7968/03	4.3.2	LAM	3.8	0.6	46
946/03	4.3.4.1	LAM	7.5	0.8	46
2253/99	4.6.1.1	Uganda	7.5	0.5	45
2169/99	4.6.1.2	Uganda	3.8	0.1	45
5390/02	4.6.2.2	Cameroon	3.8	1.0	45

a Data are the means of 3 to 6 independent experiments.

b EAI, East African Indian; LAM, Latin American Mediterranean.

Our previous work on a small number of clinical isolates suggested that wollamide B1 inhibits the growth of MDR and XDR M. tuberculosis strains (15). We expanded the clinical relevance of these initial observations by assessing wollamide B1 activity against an extended panel of clinical MTBC strains representing lineages 1, 2, 3, and 4, with single and multiple antibiotic resistances (Table 2 and 3). To accommodate the range of wollamide B1 sensitivity observed in clinical isolates (Table 1), sensitivity to the significantly less active control wollamide B3 (MIC, > 30 μM for M. tuberculosis H37Rv [15]) was used as a reference point for each MTBC strain. Compared to the control compound wollamide B3, wollamide B1 inhibited growth of 89% (31/35) of the M. tuberculosis drug-resistant isolates with MIC values 2- to 45-fold lower than those of wollamide B3 (Table 3). Together, these results demonstrate antimycobacterial activity of wollamide B1 against phylogenetically diverse clinical MTBC strains that is not compromised by common, genetically conferred antibiotic resistance mechanisms in clinical MTBC strains.

**TABLE 2:** Phenotypic and genetically conferred drug resistance of clinical M. tuberculosis complex strains^*a*^

Isolate	Phenotypic drug resistance	Genetic drug resistance (mutations)
18009945	DLM, PZA intermediate	*fbiA* (G365A)
19002407	BDQ	ND
19002408	BDQ	ND
19007331	INH, RMP, EMB, LEV, MOX, AMK	*katG* (S315T), *rpoB* (S450L), *embB* (M306I), gyrA (D94N, D94Y), *rrS* (A1401G), *rpsL* (K43R)
19008075	PZA, BDQ, CFZ	ND
19008346	INH, RMP, EMB, PZA, LEV, MXF, AMK, CAP, KAN, PTH/ETH, PAS, CYC	ND
20000168	INH, DLM	ND
20000172	INH, DLM	ND
20000173	BDQ	*atpE* (A63P)
21001264	RMP, SM	*rpoB* (H445N), *gid* (351_del_g)
20001994	ND	ND
20003074	DLM	ND
20004048	SM, INH, EMB, PZA, KAN	ND
20004521	INH, EMB, PTH/ETH, SM	*katG* (S315T), *embB* (M306V), *rpsL* (K43R), *ethA* (T314I)
20004726	INH, RMP, PZA, CAP, EMB	*katG* (S315T), *rpoB* (S450L), *embB* (M306I), *tlyA* (N236K), *pncA* (W68C)
20004924	INH, RMP, PZA, CAP	*katG* (S315T), *rpoB* (S450L), *embB* (M306I), *tlyA* (N236K), *pncA* (W68C)
20005888	INH, EMB, PZA	*katG* (S315T), *embB* (M306I), *pncA* (F81C)
20005890	SM, INH, EMB	*katG* (S315T), *embB* (M306I), *pncA* (F81C)
20006645	INH, EMB, AMI, KAN, PTH	*katG* (S315T), *fabG1* (L203L), *rpoB* (D435Y), *embB* (M306V), *rrS* (C517T), *ethA* (76S_del_g), eis-Promoter (-14c>t)
20007046	INH, RMP, EMB, PZA, MOX, AMK, CAP, KAN, PTH/ETH, SM	*katG* (S315T), *fabG1* (-15c>T), *rpoB* (S450L), *embA* (-12c>t), *embB* (Y334H), *rrS* (A1401G), *pncA* (S104R), *gyrB* (E501D), *rpsL* (K88R)
20007047	INH low, RMP, EMB, PZA, LEV, MOX, CAP, PTH/ETH, SM	*inhA* (I194T), *gyrA* (A90V), *rpoB* (S450L), *rpsL* (K43R), *fabG1* (-15C>T), *pncA* (S164P), *embB* (M306V)
21001832	INH, RMP, EMB, SM	*katG* (S315T), *rpoB* (S531L, S450L), *embB* (Q497R)
21002232	INH, RMP, PZA	*katG* (S315T), *rpoB* (S531L, S450L), *pncA* (K96T), *rpsL* (K43R)
21002399	INH low, RMP, PZA, PTH/ETH	*rpoB* (S531L, S450L), *fabG1* (-15c>T), *pncA* (H51R)
21002574	INH, RMP, LEV, MOX, BDQ, CLZ	*rpoB* (I1491F), *gyrA* (D94H), *Rv0678* (138_ins_g)
21002663	INH, RMP, EMB, PZA, SM	*katG* (S315T), *rpoB* (S450L), *embA* (-8c>a), *embB* (G406S), *rpsL* (K43R), *pncA* (-11a>g)
21002756	INH, RMP, EMB, PZA	*katG* (S315T), *rpoB* (S450L), *rrS* (514a>c), *pncA* (W68R)
21005858	INH, LEV, MOX, CAP, PTH/ETH	ND
10213-12	PA	ND
10425-15	BDQ, CFZ	ND
347-10	PA, DLM	ND
348-10	PA, DLM	ND
349-10	PA, DLM	ND
8844-10	PA	ND
9412-11	INH, EMB, AMK, CAP, KAN	ND

a M. tuberculosis isolates with confirmed resistance against INH (isoniazid), RMP (rifampicin), EMB (ethambutol), PZA (pyrazinamide), PTH (prothionamide), ETH (ethionamide), CAP (capreomycin), DLM (delamanid), SM (streptomycin), LEV (levofloxacin), MOX (moxifloxacin), AMK (amikacin), KAN (kanamycin), BDQ (bedaquiline), CFZ (clofazimine), PAS (para-aminosalicylic acid), CYC (cycloserine), PA (pretomanid). ND, not determined.

**TABLE 3:** Wollamide B1 inhibits growth of phylogenetically diverse clinical strains of M. tuberculosis complex with antibiotic resistance profiles^*a*^

Isolate	Lineage	Genotype	Resistance	Wollamide B1 MIC (μM)	Wollamide B3 MIC (μM)
10213-12	1.1.1.1	EAI	PA	30	150
8844-10	1.1.1.1	EAI	PA	30	>150
20005888	1.1.2	EAI	INH, EMB, PZA	30	150
20005890	1.1.2	EAI	SM, INH, EMB	30	75
20000168	1.1.3	EAI	INH, DLM	10	150
20000172	1.1.3	EAI	INH, DLM	10	75
19007331	2.2.1	Beijing	INH, RMP, EMB, LEV, MOX, AMK	10	30
20004048	2.2.1	Beijing	SM, INH, EMB, PZA, KAN	75	>150
21001832	2.2.1	Beijing	INH, RMP, EMB, SM	>30	>30
21002663	2.2.1	Beijing	INH, RMP, EMB, PZA	30	>150
20006645	2.2.1	Beijing	INH, EMB, AMK, KAN, PTH/ETH	30	150
20007046	2.2.1	Beijing	INH, RMP, EMB, PZA, MOX, AMK, CAP, KAN, PTH/ETH, SM	30	>30
20004521	2.2.1	Beijing	INH, EMB, PTH/ETH, SM	30	>30
20007047	2.2.1	Beijing	INH low, RMP, EMB, PZA, LEV, MOX, CAP, PTH/ETH, SM	30	75
21002574	3.1.2.1	Delhi-CAS	INH, RMP, LEV, MOX, BDQ, CFZ	30	75
20001264	3.1.2.1	Delhi-CAS	RMP, DLM	30	150
21002756	4.3.1	LAM	INH, RMP, EMB, PZA	30	150
21002399	4.3.3	LAM	INH low, RMP, PZA, PTH/ETH	10	>30
19008346	4.3.3	LAM	INH, RMP, EMB, PZA, LEV, MOX, AMK, CAP, KAN, PTH/ETH, PAS, CYC	30	>150
21002232	4.4.1.1	S-type	INH, RMP, PZA	30	150
20003074	4.5	Euro-American	DLM	75	75
20004726	4.6.2	Euro-American	INH, RMP, PZA, CAP	30	150
20004924	4.6.2	Euro-American	INH, RMP, PZA, CAP	10	75
20001994	4.6.2	Euro-American	N.D.	75	75
21005858	4.6.2	Euro-American	INH, LEV, MOX, CAP, PTH/ETH	10	150
18009945	4.8	H37Rv-like	DLM, PZA intermediate	30	>30
19002407	4.9	H37Rv-like	BDQ	30	>150
20000173	4.9	H37Rv-like	BDQ	3.3	150
347-10	4.9	H37Rv-like	PA, DLM	10	150
348-10	4.9	H37Rv-like	PA, DLM	30	150
349-10	4.9	H37Rv-like	PA, DLM	30	150
19002408	ND	ND	BDQ	30	>150
19008075	ND	ND	PZA, BDQ, CFZ	30	150
10425-15	ND	ND	BDQ, CFZ	>30	>30
9412-11	ND	ND	INH, EMB, AMK, CAP, KAN	30	>30

a M. tuberculosis isolates with confirmed resistance against INH (isoniazid), RMP (rifampicin), EMB (ethambutol), PZA (pyrazinamide), PTH (prothionamide), ETH (ethionamide), CAP (capreomycin), DLM (delamanid), SM (streptomycin), LEV (levofloxacin), MOX (moxifloxacin), AMK (amikacin), KAN (kanamycin), BDQ (bedaquiline), CFZ (clofazimine), PAS (para-aminosalicylic acid), CYC (cycloserine), PA (pretomanid). EAI, East African Indian; LAM, Latin-American Mediterranean; CAS, central Asian; ND, not determined.

### Wollamide B1 synergizes with delamanid, pretomanid, ethambutol, and para-aminosalicylic acid in inhibiting growth and survival of M. tuberculosis *in vitro*.

We previously showed synergy between wollamide B1 and pretomanid in inhibiting growth of the M. tuberculosis reference strain H37Rv (15). This encouraged more detailed interrogation of the potential synergistic profile of the wollamide pharmacophore with clinically relevant TB antibiotics. To identify TB antibiotics that synergize with wollamides, but also potential antagonism of currently used antibiotics by wollamides, we employed an *in vitro* broth dilution checkerboard method to calculate the fractional inhibitory concentration index (FICI) (20) and zero interaction potency (ZIP) scores (21).

**FIG 1:**
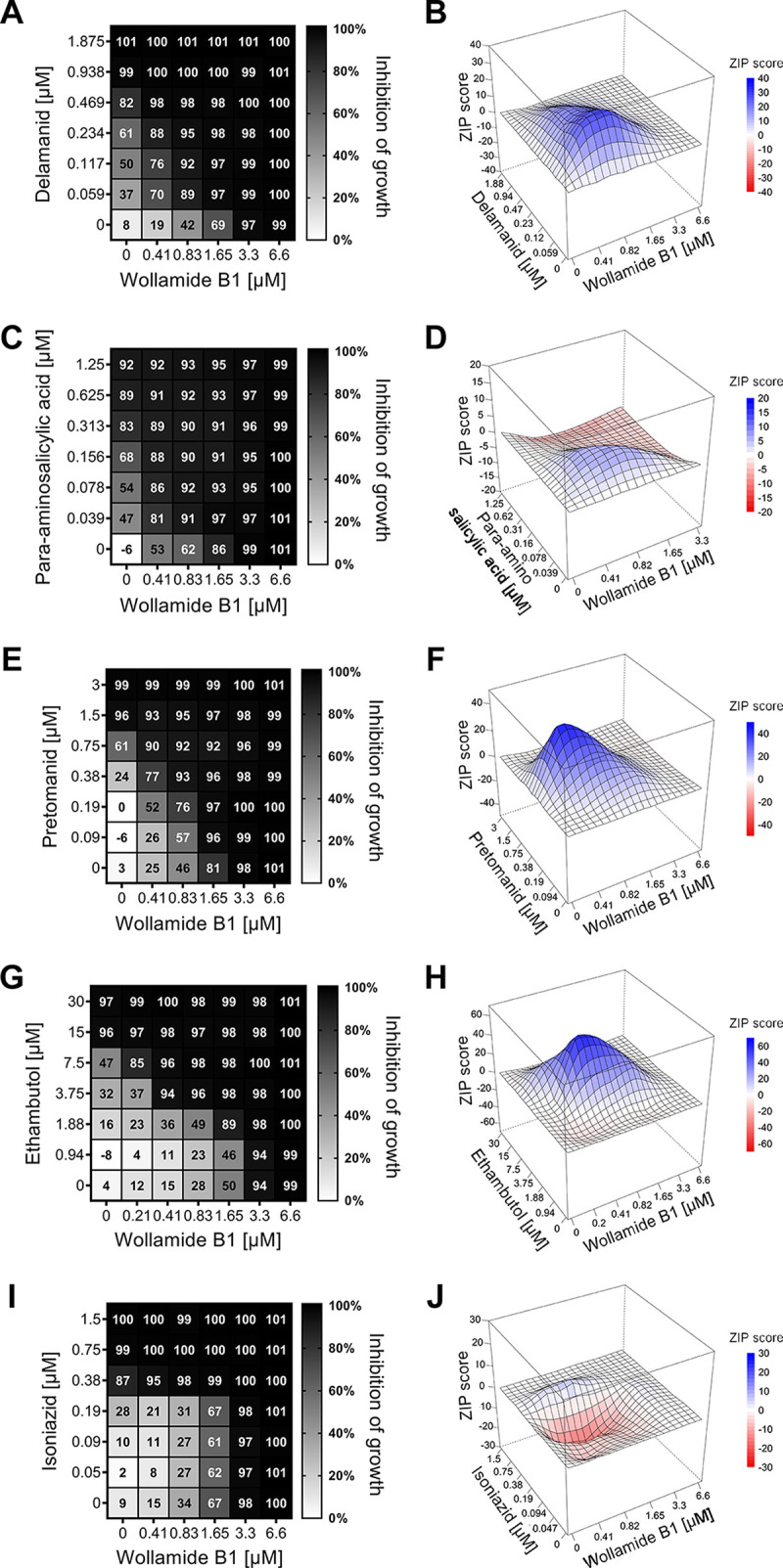
(A to J) Wollamide B1 synergizes with delamanid (A and B), para-aminosalicylic acid (C and D), pretomanid (E and F), and ethambutol (G and H), but not with isoniazid (I and J) in the growth inhibition of M. tuberculosis H37Rv in liquid culture. (A, C, E, G, and I) Checkerboards demonstrate shifts in the percent inhibition of growth of M. tuberculosis H37Rv by combinations of wollamide B1 and the drug of interest. (B, D, F, H, and J) Three-dimensional (3D) interaction profiles show synergistic peaks upon combinations of wollamide B1 and the drug of interest. Zero interaction potency (ZIP) scores represent the percent response beyond the expected response due to drug interactions (21). The data represent mean of 4 to 7 independent cultures analyzed in 3 to 4 independent experiments.

We analyzed the interactions between wollamide B1 and antibiotics used for the treatment of TB in the growth inhibition of the M. tuberculosis reference strain H37Rv (Table 4). A total of 12 antibiotics, including isoniazid, rifampicin, bedaquiline, linezolid, and moxifloxacin, displayed indifferent interactions with wollamide B1 (Table 4). The interaction between streptomycin and wollamide B1 indicated antagonism, based on the FICI value (FICI, 5.0), but not the ZIP score (ZIP, 1) (Table 4). In contrast, synergistic interactions between wollamide B1 and ethambutol (FICI, 0.38; ZIP, 58), delamanid (FICI, 0.19; ZIP, 26), para-aminosalicylic acid (FICI, 0.28; ZIP, 10) were identified, and synergism with pretomanid confirmed (FICI 0.31, ZIP 43) (15) (Table 4; Fig. 1). A lower degree of synergy was also observed between wollamide B1 and ethionamide (FICI, 0.38; ZIP, 6) and levofloxacin (FICI, 0.5; ZIP, 6), where the FICI score indicated synergy (≤0.5), but the ZIP score did not (<10) (Table 4). Synergism between wollamide B1 extended to phylogenetically diverse clinical MTBC strains, where all isolates demonstrated synergy with delamanid, 6 of 7 demonstrated synergy with ethambutol, and 5 of 7 demonstrated synergy with pretomanid or para-aminosalicylic in inhibiting mycobacterial growth (FICI, <0.5; Table 5).

**TABLE 4:** Wollamide B1 synergism with select tuberculosis antibiotics in inhibiting the growth of M. tuberculosis H37Rv^*a*^

Agent	Class	Target	Mechanism of action	MIC (μM)	FICI	ZIP score	Reference
Wollamide B1	Cyclic hexapeptide	Unknown	Unknown	3.3	NA	NA	14, 15
Delamanid	Nitroimidazole	Unknown	Mycolic acid synthesis; respiratory poisoning	0.9	**0.19**	**26**	47, 48
Para-amino salicylic acid	Aminobenzoic acid	Dihydrofolate reductase	Folic acid synthesis; iron metabolism	0.6	**0.28**	**10**	49
Pretomanid	Nitroimidazole	Unknown	Mycolic acid synthesis; respiratory poisoning	1.5	**0.31**	**43**	24, 48, 50
Ethambutol	ND	Arabinosyl transferases	Arabinogalactan synthesis	15	**0.38**	**58**	23, 51
Ethionamide	Thionamide	Enoyl-acyl carrier protein InhA	Mycolic acid synthesis	60	**0.38**	6	52, 53
Levofloxacin	Fluoroquinolone	DNA gyrase and DNA topoisomerase I	DNA synthesis	1.4	0.5	6	54
Isoniazid	Hydrazide	Enoyl-acyl carrier protein InhA	Mycolic acid synthesis; respiratory poisoning	0.8	0.75	8	55, 56
Cycloserine	d-Alanine analog	d-Alanine racemase and ligase	Peptidoglycan synthesis	6.3	2.0	1	57
Imipenem	Beta-lactam	Penicillin-binding proteins	Peptidoglycan synthesis	100	2.0	ND	58
Bedaquiline	Diarylquinoline	F1F0-ATP synthase	ATP synthesis (respiration/oxidative phosphorylation)	0.9	2.0	2	59 – 61
Moxifloxacin	Fluoroquinolone	DNA gyrase and DNA topoisomerase I	DNA synthesis	0.9	2.0	1	54, 62
Rifampicin	Rifamycin	RNA polymerase, beta subunit	RNA synthesis	0.2	2.0	2	63
Amikacin	Aminoglycoside	30S ribosomal subunit	Protein synthesis	2.5	2.0	2	64 – 66
Linezolid	Oxazolidinone	23S rRNA of 50S ribosomal subunit	Protein synthesis	2.5	2.0	4	62, 67, 68
Niclosamide	Benzamide	AR-V7	Membrane potential; pH homeostasis	15	3.0	2	69
Capreomycin	Cyclic polypeptide	Interbridge 2a between 30S and 50S ribosomal subunits	Protein synthesis	0.4	3.0	0	70, 71
Kanamycin	Aminoglycoside	30S ribosomal subunit	Protein synthesis	6.3	3.0	0	64, 66, 72
Streptomycin	Aminoglycoside	S12 and 16S rRNA of 30S ribosomal subunit	Protein synthesis	1.9	5.0	1	66, 70

a FICI, fractional inhibitory concentration index; ≤0.5, synergy; 0.5≤4.0 indifference; >4.0 antagonism. ZIP, zero interaction potency score; >10, synergy; <–10, antagonism. Bold denotes synergistic interactions. ND, not determined.

**TABLE 5:** Wollamide B1 synergizes with ethambutol, pretomanid, delamanid and para-aminosalicylic acid in the growth inhibition of phylogenetically diverse clinical isolates of the M. tuberculosis complex^*a*^

Isolate	Lineage	Genotype	Delamanid	Para-amino salicylic acid	Pretomanid	Ethambutol
H37Rv	4	Euro-American	**0.19**	**0.28**	**0.31**	**0.38**
4130/02	4.1.2.1	Haarlem	**0.13**	**0.28**	**0.31**	**0.28**
946/03	4.3.4.1	LAM	**0.25**	**0.24**	**0.40**	**0.48**
2169/99	4.6.1.2	Uganda	**0.31**	**0.09**	**0.13**	**0.19**
4850/03	1.1.2	EAI	**0.25**	**0.26**	0.56	**0.31**
11406/08	1.2.1	EAI	**0.09**	1.00	0.75	**0.28**
1500/03	2.2.1	Beijing modern	**0.28**	**0.38**	**0.16**	0.53
57/02	2.2.2	Beijing ancestral	**0.09**	0.51	**0.38**	**0.28**

a FICI, fractional inhibitory concentration index, a score which evaluates the interaction of two compounds. ≤0.5, synergy; 0.5≤4.0 indifference; >4.0 antagonism. The data represent the mean of FICI scores from 3 to 4 independent experiments. EAI, East African Indian; LAM, Latin-American Mediterranean. Bold denotes synergistic interactions.

**FIG 2:**
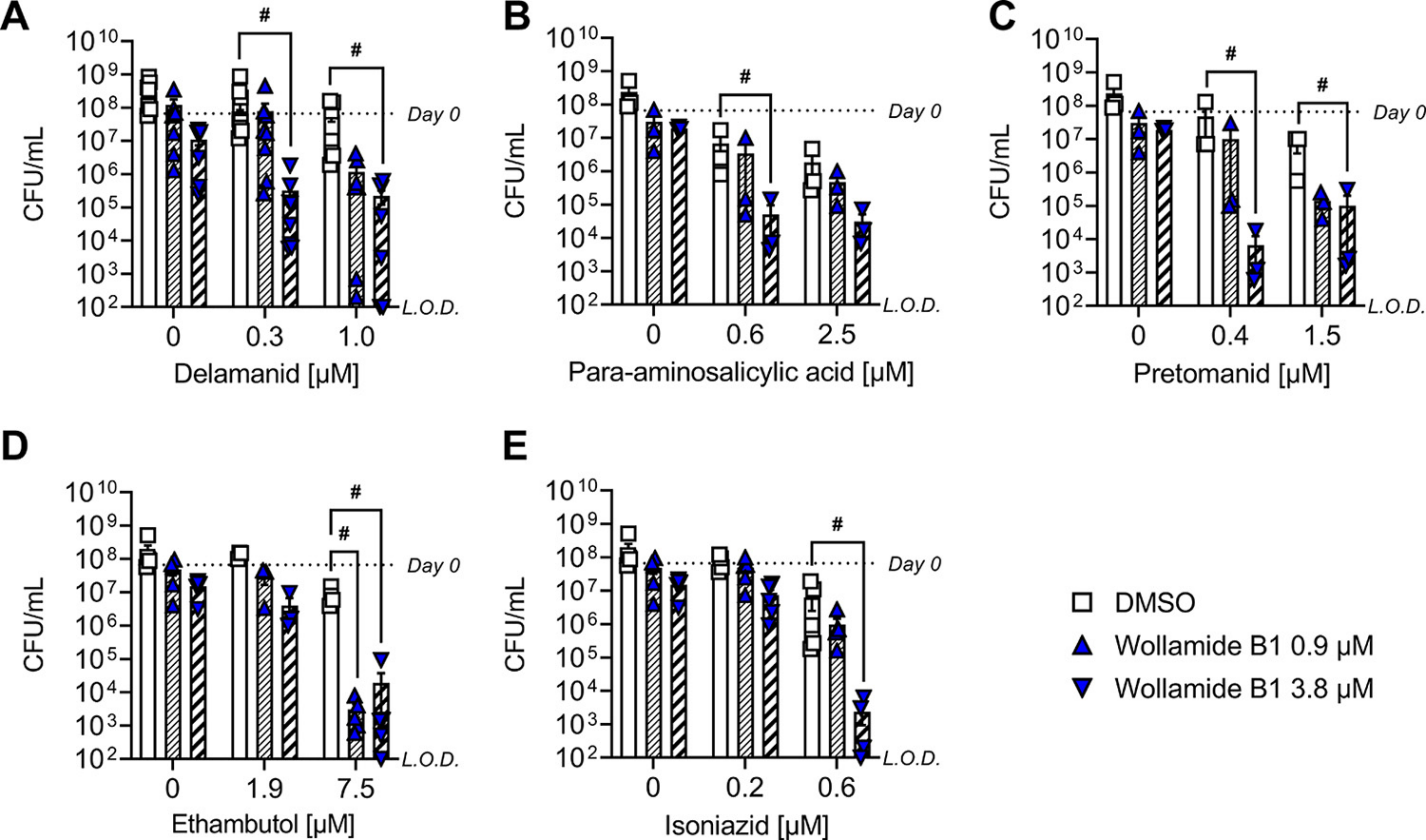
(A to E) Wollamide B1 synergizes with (A) delamanid, (B) para-aminosalicylic acid, (C) pretomanid, (D) ethambutol, and (E) isoniazid in bactericidal activity against M. tuberculosis H37Rv. The addition of wollamide B1 to (A) delamanid, (B) para-aminosalicylic acid, (C) pretomanid, (D) ethambutol, and (E) isoniazid amplified bactericidal activity compared to the drug alone after 10 days of incubation. The data are the mean ± SEM of 3 to 8 independent cultures across three independent experiments. L.O.D., limit of detection at 10^2^ CFU/mL; #, synergistic interaction of wollamide B1 and antibiotic defined as a decrease in CFU/mL greater than 2 × log_10_ compared to antibiotic alone (22).

We also assessed the effect of wollamide B1 on the bactericidal activity of TB antibiotics. We confirmed that wollamide B1 exerted bactericidal activity against M. tuberculosis H37Rv liquid cultures, significantly reducing the CFU/mL after 3, 6, and 10 days of incubation with 3.8 μM wollamide B1 (mixed effects analysis with Tukey’s multiple corrections, *P* < 0.05). We then demonstrated that wollamide B1 amplified the bactericidal activity of ethambutol, delamanid, pretomanid, and para-aminosalicylic acid, where the addition of 3.8 μM wollamide B1 reduced the CFU/mL of the combination by more than 2 × log_10_ compared to the antibiotic alone (22) (Fig. 2; Table 6). Moreover, wollamide B1 also increased the bactericidal activity of isoniazid (Fig. 2; Table 6).

**TABLE 6:** M. tuberculosis H37Rv viability after 10 days of exposure to wollamide B1 and combinations of TB antibiotics in liquid cultures^*a*^

Compound	Antibiotic (μM)	log10 CFU/mL		log10 CFU/mL compared to DMSO	Δlog10 CFU/mL compared to most active single agent
+ DMSO	DMSO	8.3	±0.1		
	DLM 0.3	7.8	±0.2	−0.5	
	DLM 1.0	7.3	±0.3	−1.0	
	PA 0.4	7.3	±0.4	−1.1	
	PA 1.5	6.6	±0.4	−1.8	
	EMB 1.9	8.1	±0.1	−0.2	
	EMB 7.5	6.9	±0.1	−1.5	
	PAS 0.6	6.5	±0.4	−1.8	
	PAS 2.5	5.9	±0.4	−2.4	
	INH 0.2	7.8	±0.1	−0.5	
	INH 0.6	6.2	±0.4	−2.2	
+ Wollamide B1 (0.9 μM)	DMSO	7.6	±0.3	−0.7	
	DLM 0.3	7.1	±0.4	−1.2	−0.5
	DLM 1.0	5.7	±0.3	−2.7	−1.7
	PA 0.4	5.9	±0.8	−2.5	−1.4
	PA 1.5	5.0	±0.2	−3.3	−1.5
	EMB 1.9	7.3	±0.4	−1.1	−0.4
	EMB 7.5	3.3	±0.2	−5.0	**−3.6**
	PAS 0.6	5.6	±0.7	−2.7	−0.9
	PAS 2.5	5.9	±0.4	−2.4	0.0
	INH 0.2	7.6	±0.2	−0.8	−0.1
	INH 0.6	5.8	±0.2	−2.5	−0.4
+ Wollamide B1 (3.8 μM)	DMSO	6.5	±0.3	−1.8	
	DLM 0.3	4.2	±0.5	−4.2	**−2.3**
	DLM 1.0	4.2	±0.6	−4.2	**−2.3**
	PA 0.4	3.4	±0.5	−5.0	**−3.1**
	PA 1.5	4.0	±0.7	−4.3	**−2.5**
	EMB 1.9	6.4	±0.3	−2.0	−0.1
	EMB 7.5	3.2	±0.5	−5.1	**−3.3**
	PAS 0.6	4.2	±0.5	−4.1	**−2.3**
	PAS 2.5	4.3	±0.3	−4.0	−1.6
	INH 0.2	6.7	±0.2	−1.7	0.2
	INH 0.6	2.9	±0.4	−5.4	**−3.3**

a Data are log_10_ CFU/mL at day 10. Δlog_10_ CFU indicates the reduction in CFU/mL of the combination compared to the most active single agent (22). Data are the means ± SEM of 3 to 8 independent cultures from 3 independent experiments. Bold denotes Δlog_10_ of < –2.0, i.e., synergistic interaction as defined by reference 22. DLM, delamanid; PA, pretomanid; EMB, ethambutol; PAS, para-aminosalicylic acid; INH, isoniazid.

In contrast to wollamide B1, the cyclic peptide aminoglycoside capreomycin did not synergize with delamanid, ethambutol, para-aminosalicylic acid, or pretomanid (Table 7), suggesting selectivity in the synergism between these antibiotics and the cyclic peptide wollamide B1.

**TABLE 7:** Capreomycin does not synergize with ethambutol, pretomanid, delamanid, or para-aminosalicylic acid in inhibiting the growth of M. tuberculosis H37Rv in liquid culture^*a*^

Compound	Wollamide B1	Capreomycin
Wollamide B1	NA	3.0
Delamanid	**0.19**	2.0
Para-aminosalicylic acid	**0.28**	2.0
Pretomanid	**0.31**	0.75
Ethambutol	**0.38**	2.0
Isoniazid	0.75	1.0

a Data represent the mean of FICI scores obtained in 3 to 4 independent experiments: ≤0.5, synergy; 0.5 ≤ 4.0, indifference; >4.0, antagonism. Bold denotes synergistic interactions.

Taken together, these data demonstrate that wollamide B1 synergizes with current antibiotics that are cornerstones of TB treatment regimens for drug-sensitive and drug-resistant TB.

### Wollamide B1 does not antagonize standard tuberculosis antibiotic combinations and enhances M. tuberculosis susceptibility to the combination of bedaquiline, pretomanid, and linezolid.

**FIG 3:**
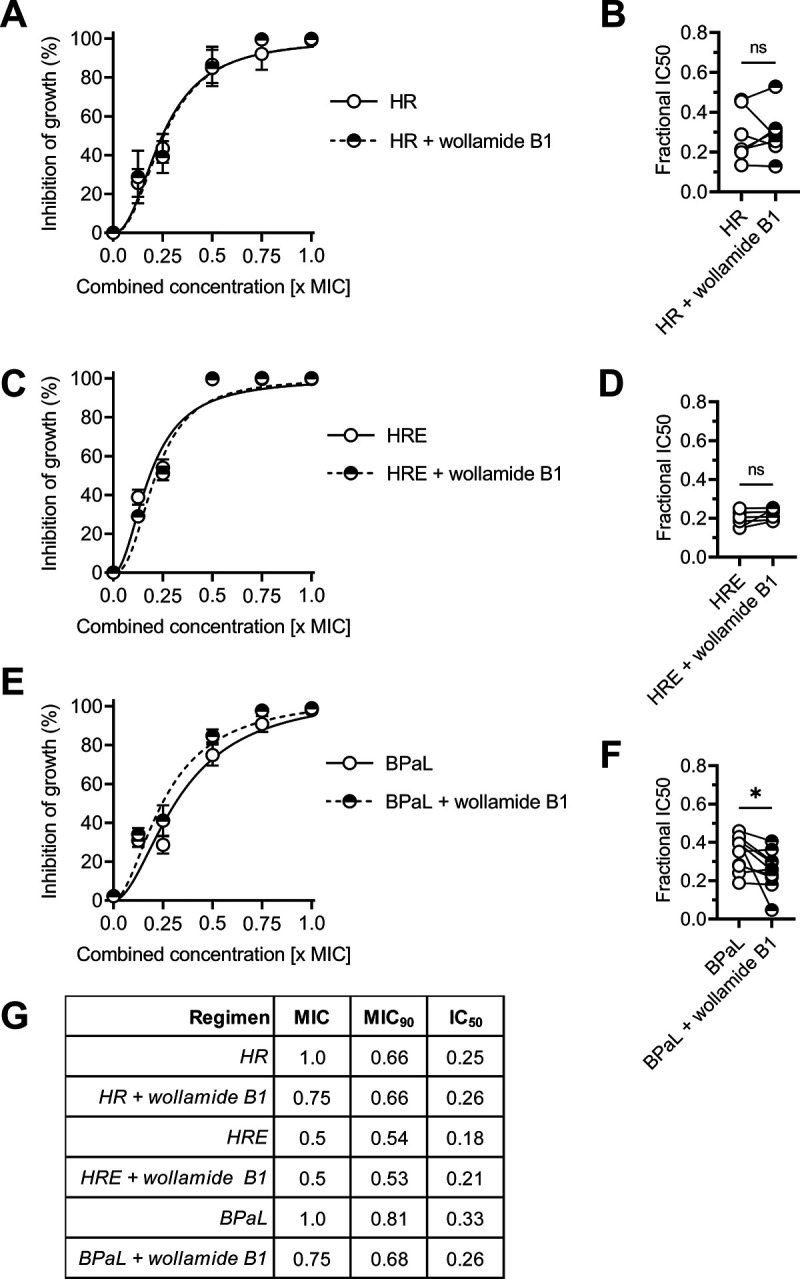
Wollamide B1 enhances the inhibition of M. tuberculosis H37Rv growth by the combination of bedaquiline/pretomanid/linezolid. (A, C, and E) Percent inhibition of M. tuberculosis H37Rv growth in liquid culture in the presence of (A) isoniazid (H) and rifampicin (R), (C) isoniazid (H), rifampicin (R), and ethambutol (E), (E) bedaquiline (B), pretomanid (Pa), and linezolid (L), with and without wollamide B1. Data are the mean ± SEM of 10 independent cultures across 5 independent experiments. (B, D, and F) Fractional IC_50_ of antibiotic combinations with and without wollamide B1 extracted from 10 independent cultures across 5 independent experiments. (A, C, and E) Paired Student’s *t* test; *, *P* < 0.05. (G) MIC, MIC_90_, and IC_50_ (as fractions of the concentration [μM] of individual agents) of inhibition of M. tuberculosis H37Rv growth in liquid culture by antibiotic combinations with and without wollamide B1 as the means of *n* = 10 independent cultures across 5 independent experiments (as shown in panels A, C, and E).

Wollamide B1 synergized with select TB antibiotics and did not antagonize most other antibiotics tested, including the key first-line drugs isoniazid and rifampicin (Table 4). We therefore assessed how wollamide B1 interacts with TB treatment regimens. Wollamide B1 lowered the fractional inhibitory MIC of the combination of isoniazid (H) and rifampicin (R) without affecting the 50% inhibitory concentration (IC_50_; H/R MIC = 1.0, IC_50_ = 0.25; H/R + wollamide B1 MIC = 0.75, IC_50_ = 0.26) (Fig. 3A, B, and G). Addition of ethambutol (E), an antibiotic with which wollamide B1 synergizes (Table 4), did not further enhance growth inhibition by the H/R/wollamide B1 combination (Fig. 3C, D, and G). Addition of wollamide B1 to the recently approved combination of bedaquiline (B), pretomanid (Pa), and linezolid (L) (1, 8) led to a reduction of both the fractional MIC (BPaL, 1.0; BPaL + wollamide B1, 0.75) and the IC_50_ (BPaL, 0.33; BPaL + wollamide B1, 0.26; Student’s *t* test *P* = 0.03) (Fig. 3E, F, and G).

Thus, wollamide B1 moderately enhances the antimycobacterial activity of the BPaL regimen and does not antagonize *in vitro* antimycobacterial activity of the H/R(±E) combinations.

## DISCUSSION

The discovery of new compounds with antimicrobial activity can foster the development of new TB antibiotics urgently called for by the World Health Organization and other agencies (1). Here, we show that wollamide B1, a synthetic analogue of the natural product wollamide cyclic hexapeptide (14), exhibits antimycobacterial activity against phylogenetically diverse clinical MTBC strains, including multi- and extensively drug resistant isolates. Wollamide B1 synergizes with clinically relevant first- and second-line TB antibiotics, enhances antimycobacterial activity of the recently approved BPaL regimen, and does not antagonize the major first-line TB treatment regimen of isoniazid and rifampicin.

Wollamide B1 synergizes with antibiotics that inhibit the synthesis of components unique to mycobacterial cell walls, such as arabinogalactan (ethambutol [23]) and mycolic acids (pretomanid [24], delamanid [25], and to a lesser extent, ethionamide [26] and isoniazid [27]) (Table 4). These findings extend our knowledge of antimycobacterial agents that synergize with pretomanid, thus far including rifampicin, bedaquiline, ethambutol, isoniazid, and ethionamide (28–30); ethambutol has demonstrated synergy with bedaquiline, pretomanid, and moxifloxacin (29, 31). In contrast, little is known about how delamanid and para-aminosalicylic acid interact with other antibiotics, and a prediction algorithm suggested no synergy with known TB antibiotics (29). These findings encourage interrogation of the molecular mechanisms underpinning synergism between wollamide B1 and these antibiotics with a view to enhancing their efficacy, reducing toxicity, and slowing drug resistance. Investment in delineating the wollamide mechanism of action will be invaluable in this pursuit. Wollamides do not exhibit activity against Gram-negative bacteria and, compared to M. tuberculosis, have comparatively low activity against Gram-positive bacteria (14, 19). The latter is not mediated by nonspecific disruption of bacterial membrane potential (19). Whether wollamides have a distinct (mycobacterial-)specific molecular target or exert nonspecific perturbations of the mycobacterial cell wall remains to be elucidated. Either possibility could account for the synergism between tuberculosis antibiotics in killing mycobacteria. Any mechanistic model of interactions between wollamides and TB antibiotics will need to explain the relative selectivity of wollamide synergism with antibiotics that target mycobacterial cell wall synthesis via diverse mechanisms, rather than “promiscuous” synergizing properties with antibiotics that inhibit bacterial synthesis of proteins, DNA, RNA, peptidoglycan, or ATP (Table 4) (32, 33).

Complex antibiotic regimens are designed for superior anti-bacterial activity compared to single drugs, exert multiple mechanisms of action across different molecular targets, and aim to prevent or delay emergence of bacterial drug resistance (34). Higher-order drug combinations often display weaker synergy compared to pairwise comparisons (28), which is reflected by our observations on wollamide B1 addition to the HRE and BPaL regimens compared to their pairwise interactions. Future work will be required to explore whether a wollamide-inspired antimycobacterial agent can enhance activity of TB drug regimens, including BPaL, *in vivo.* This will require further investment in structure-activity-guided optimization of the wollamide scaffold for enhanced *in vivo* bioavailability (15, 17) or a rationally designed inhibitor of the wollamide B1 molecular target, which remains to be reported.

The present study expands our knowledge of the beneficial properties of the wollamide pharmacophore, including activity against phylogenetically diverse M. tuberculosis across major MTBC lineages, including MDR and XDR clinical isolates (Tables 1 and 3), activity against replicating and nonreplicating M. tuberculosis (Fig. 2) (15), activity against intracellular mycobacteria (14, 15), and low mammalian cytotoxicity (15–19). Probing synergistic interactions between wollamide B1 with antibiotics and selective inhibitors (e.g., of cell wall synthesis) will be invaluable for the interrogation of the molecular mechanisms underpinning the wollamide antimycobacterial activity. Such insights might be exploited for enhancing the efficacy of TB treatment regimens and limiting the toxicity of current treatments by reducing the duration and doses required for clinical efficacy.

## MATERIALS AND METHODS

### Bacteria, antibiotics, and wollamides.

Mycobacterium tuberculosis strain H37Rv (NR-13648) was obtained through BEI Resources, NIAID, NIH. Pan-susceptible M. tuberculosis clinical isolates are summarized in Table 1. Drug-resistant M. tuberculosis clinical isolates were selected from the collection at the National and WHO Supranational Reference Center for Mycobacteria, Leibniz Lung Center, Borstel, Germany (Table 2).

Commercially available antibiotics were purchased: isoniazid, rifampicin, moxifloxacin, amikacin, kanamycin, niclosamide, pretomanid, 4-aminosalicylic acid (para-aminosalicylic acid), streptomycin sulfate (Sigma-Aldrich); linezolid, cycloserine, capreomycin, ethionamide, levofloxacin (Cayman Chemical); delamanid, bedaquiline (Selleck Chemicals); imipenem (AdooQ Bioscience); ethambutol hydrochloride (European Pharmacopoeia Reference Standard). Isoniazid, moxifloxacin, ethambutol, capreomycin, amikacin, kanamycin, streptomycin, and imipenem were dissolved in sterile distilled water; all other antibiotics were dissolved in sterile dimethyl sulfoxide (DMSO) (Sigma-Aldrich).

Wollamides B1 and B3 were synthesized in a similar manner to our previous report (15). Briefly, peptides were synthesized on 2-chlorotrityl resin (0.8 mmol/g; Chem Impex) at a 75-μM scale on a Symphony multiplex synthesizer. Amino acids (5 eq.) were activated using 2-(6-chloro-1H-benzotriazole-1-yl)-1,1,3,3-tetramethylaminium hexafluorophosphate (HCTU) (5 eq) and DIPEA (*N*,*N*-diisopropylethylamine) (10 eq) in dimethylformamide (DMF) (2 × 30 min), prior to removing the N-terminal Fmoc (9-fluorenylmethoxy carbonyl) protecting group using 20% piperidine in DMF (2 × 5 min). Protected peptides were cleaved from resin by adding a solution of 2% trifluoroacetic acid (TFA) in dichloromethane (DCM) into a pyridine-water-MeOH solution (5 × 2 min). Concentrating the mixture under reduced pressure and adding water gave a white precipitate, which was collected by centrifugation and dissolved in 50:50 acetonitrile:water. The protected linear peptide was then freeze-dried and cyclized without further purification. Cyclization of the protected linear peptide was conducted at 1.5 mM concentration in DMF with 2 equivalents of BOP ((benzotriazol-1-yloxy)tris(dimethylamino)phosphonium hexafluorophosphate) and DIPEA, respectively. The DMF was removed under vacuum, and the cyclic peptide was treated with 10 mL TFA:TIPS(trisopropylsilane):H_2_O (95:2.5:2.5) over 2 h. The solutions were then dried under nitrogen, and the resulting materials were precipitated with ice-cold diethyl ether and lyophilized. Peptides were purified to >95% purity using a reversed-phase Shimadzu high-pressure liquid chromatography (HPLC) system; Phenomenex C_18_ 10 μM, 100-Å, 250 × 21.2-mm semipreparative column; 20 mL/min; isocratic 0% solvent B (H_2_O:MeCN 10:90 with 0.1% TFA) in solvent A (H_2_O with 0.1% TFA) over 5 min and gradient 0% to 70% solvent B in solvent A over 50 min. Purified fractions were freeze-dried to give a white powder.

### Bacterial culture.

M. tuberculosis was grown at 37°C in 7H9 Middlebrook medium (Becton Dickinson Biosciences) with 0.2% (vol/vol) glycerol (Sigma-Aldrich) and 0.05% (vol/vol) Tween 80 (Sigma-Aldrich), supplemented with either 10% albumin-dextrose-NaCl (ADN; 5% [wt/vol] bovine serum albumin fraction V [BSA; Roche], 2% [wt/vol] glucose [Sigma-Aldrich], 0.85% [vol/vol] NaCl [Thermo Fisher Scientific]) or 10% oleic acid-albumin-dextrose-catalase (OADC; 5% [wt/vol] bovine serum albumin fraction V [BSA; Roche], 2% [wt/vol] glucose [Sigma-Aldrich], 0.85% [vol/vol] NaCl [Thermo Fisher Scientific], 0.056% [vol/vol] oleic acid [Sigma-Aldrich], 4% [wt/vol] catalase [Sigma-Aldrich]). M. tuberculosis H37Rv was grown in 7H9 medium supplemented with 10% ADN, while clinical isolates were grown with 10% OADC supplement.

### MIC determination.

For MICs for M. tuberculosis H37Rv and pan-susceptible M. tuberculosis clinical isolates, wollamide B1 and antibiotics were 2-fold serially diluted in flat-bottom 96-well plates. Medium only, solvent (DMSO, <1% [vol/vol], or H_2_O) only, and isoniazid (20 μg/mL) were used as controls. M. tuberculosis H37Rv and pan-susceptible clinical isolates were grown to the mid-log phase (optical density at 600 nm [OD_600_], 0.4 to 0.6) in supplemented (10% ADN for H37Rv, 10% OADC for clinical isolates) 7H9 liquid medium, diluted to an OD_600_ of 0.02 in supplemented 7H9 medium, and then added to antimicrobials to a final volume of 200 μL per well. Cultures were incubated at 37°C in 5% CO_2_, and bacterial growth was assessed by measuring the OD_600_ after 10 days. The percent inhibition of growth exhibited by each antimicrobial compound was compared to the medium-only control (as maximum growth) and isoniazid (as minimum growth):

$$\%\;\text{inhibition of growth}\;=\;100\;\times \left(1\;-\left(\frac{{\text{sample OD}}_{600}-{\text{minimum OD}}_{600}}{{\text{maximum OD}}_{600}-{\text{minimum OD}}_{600}}\right)\right)$$

The MIC was determined as the lowest concentration of antimicrobial compound resulting in at least 95% inhibition of bacterial growth.

For MIC determination against drug-resistant M. tuberculosis clinical isolates, wollamide B1 and wollamide B3 were 3-fold serially diluted in round-bottom 96-well plates. Bacterial cultures, diluted to 1/200 of 0.5 McFarland standards, were added to a final volume of 200 μL per well. Medium only and DMSO only were used as controls. Cultures were incubated at 37°C in 5% CO_2_, and bacterial growth was determined via visual inspection of pellicles after 7 to 8 days. The MIC was determined as the lowest concentration of compound resulting in no growth (compared to controls) after 7 to 8 days.

### Antibiotic combinations.

Combinations of wollamides and antibiotics were tested using an *in vitro* broth dilution checkerboard method (35). Synergistic or antagonistic interactions were determined using the fractional inhibitory concentration index (FICI) score (20) and the zero interaction potency (ZIP) model (21, 36, 37). The FICI score was calculated as

$$\text{FICI}\;=\frac{\mathrm{MI}C_{\text{AB}}}{\mathrm{MI}C_{\text{A}}}+\frac{\mathrm{MI}C_{\text{BA}}}{\mathrm{MI}C_{\text{B}}},$$

where A and B are two antimicrobial agents, and AB/BA indicates the MIC of the agents when combined. For two-way interactions, test results were interpreted as follows: synergism, FICI ≤0.5; indifference/noninteraction, FICI 0.5 ≤ 4.0; antagonism, FICI >4.0 (20, 38–40). The ZIP model calculates a ZIP score as a measure of the deviation from the expectation that there is no interaction between two agents (21): synergism, ZIP ≥10; indifference/noninteraction, ZIP −10 < 10; antagonism, ZIP ≤−10 (21, 36, 37).

For assessment of interactions between three or more antimicrobials, we used the DiaMOND method (28, 41). Antimicrobials were combined in a 1:1:1(:1) mixture across a linear concentration range. The percent inhibition of bacterial growth was calculated as described and normalized to the DMSO solvent control, and the MIC or IC_50_ when combined was reported as fractions of the MIC or IC_50_ of individual agents.

### Bactericidal activity

To determine the bactericidal activity of wollamide B1 in combination with antibiotics, M. tuberculosis H37Rv single-cell suspensions were prepared by pelleting the bacterial cultures and washing the bacterial cultures with phosphate-buffered saline (PBS) plus 0.05% Tween 80 at 130 × *g* for 10 min at room temperature, prior to diluting to an OD_600_ of 0.02 in 7H9 medium supplemented with 10% ADN. Bacteria were cultured for 10 days in 96-well plates at 37°C in a humidified incubator (5% CO_2_) in the presence of individual or combinations of antimicrobials: wollamide B1 (0.9 μM or 3.8 μM), delamanid (0.3 μM or 1 μM), para-aminosalicylic acid (0.6 μM or 2.5 μM), pretomanid (0.4 μM or 1.5 μM), ethambutol (1.9 μM or 7.5 μM), or isoniazid (0.2 μM or 0.6 μM). Bacteria exposed to solvent only (DMSO or H_2_O) or medium only served as negative controls; bacteria exposed to isoniazid (20 μg/mL) served as a positive control. On day 10, serial dilutions of bacterial cultures were plated on Middlebrook 7H10 agar (Becton, Dickinson Biosciences) supplemented with 0.5% (vol/vol) glycerol (Sigma-Aldrich) and 10% OADC and incubated at 37°C for 2 to 3 weeks, and colonies were enumerated to calculate the CFU per mL. Synergistic effects of antimicrobials were defined as a reduction of at least 2 × log_10_ CFU/mL in the combination compared to the most active single antimicrobial (22).

### Data analyses.

Data are presented as the means ± standard error of the mean (SEM) of at least three independent experiments. MIC values were calculated as the lowest concentration of antimicrobial compound resulting in at least 95% inhibition of bacterial growth. IC_50_ values were calculated using GraphPad Prism software (v9.3.1), using the (inhibitor) versus response – variable slope (4 parameters). Two groups were compared by paired Student’s *t* test, multiple groups were compared by one-way analysis of variance (ANOVA) or mixed-effects analysis with Tukey’s correction for multiple comparisons. *P* values of <0.05 were considered statistically significant. Statistically significant synergy was determined via the described FICI scores (for inhibition of growth) or a log_10_ CFU/mL increase in killing greater than 2 between the combination and the most active single agent (22). ZIP scores were calculated using the synergyfinder R package (37); the functions used were ReshapeData() with the following arguments modified from base: data_type = inhibition, noise = TRUE, seed = 1; CalculateSynergy() was used with the following arguments modified from base: method = ZIP, correct_baseline = part.

### Data visualization.

ZIP synergy scores were visualized using the synergyfinder R package (37); the functions used were PlotSynergy() with the following arguments modified from base: type = 3D, method = ZIP.

### Data availability.

The raw data can be found at https://github.com/rachelrollo/wollamide-synergy.git.
